# Meigs and Mason (Ohio) Academy of Medicine

**Published:** 1871

**Authors:** A. L. Knight, T. Curtis Smith

**Affiliations:** President; Secretary


### *Meigs and Mason (Ohio) Academy of Medicine.*

[A. L. Knight, M.D., President. T. Curtis Smith, M.D., Secretary.]

### DISCUSSION ON THE USE OF THE FORCEPS.

The Academy convened at Pomeroy, Ohio, May 18th, at 7 o'clock, P. M., the President in the chair.

Dr. C. A. Barlow read a paper on the conditions requiring the use of the obstetric forceps. Passing by the history and various forms, modes of application, use and abuses of forceps, his paper states : "That taking it for granted that every well-informed practitioner admits that, when dexterously used in judiciously selected cases, the forceps are among the indispensable equipments of the humane and successful accoucher. There are ten conditions requiring their use, viz : 1. Insufficient action of the uterus or powerless labor ; 2. Exhausted uterus ; 3. Where the fœtal head is larger or more solid from ossification than usual, or the pelvis narrowed below the average; 4. Pelvis narrowed at superior strait not to exceed three or three and three-quarter inches ; 5. Inferior strait contracted ; 6. Face presentations ; 7. Head arrested after body of fœtus is delivered ; 8. Hæmorrhage, unavoidable or accidental ; 9. Puerperal convulsions ; 10. Tumors and accidents."

Cases bearing on or illustrating each head were given, and forceps directed to be applied with reference to the maternal pelvis without regard to position of fœtal head. In the use of the forceps the pelvic curves should alone be considered, and no regard be paid to the position of the head. We mean the blades of the forceps should always be passed along the sides of the pelvis, with the curves of the forceps corresponding to the curves of the maternal pelvis, and never regarding the fœtal head as other than a round body to be laid hold of by the forceps. In fact, in the large majority of cases the position of the head cannot be known or made out at the time when the forceps are demanded, because the head is tightly wedged in, the bones overlap, the integument is swollen, congested and infiltrated, entirely preventing a recognition of the fontanelles.

Dr. C. R. Reed agreed generally with the essayist ; thought inertia and exhaustion practically the same as a cause for the use of the forceps ; thought it hardly good practice to carry forceps in attending obstectric cases, but that they should be applied before mother or child were endangered by exhaustion or pressure. Had recently applied external pressure, as recommended by Dr. Playfair, of London, with advantage; thought it a good means of expediting labor, and would bring some difficult case to a good termination without forceps, when they would otherwise be required.

Dr. J. Q. A. Hudson approved the paper. Thought a country practitioner should carry his forceps with him. The forceps should be used early, or not to allow exhaustion or an opportunity for vesico-vaginal or vagino-rectal fistulæ, to be produced as a consequence of pressure before resorting to them. Thought tonie contraction of the uterus from uses of ergot, when endangering the child, an indication for their use.

Dr. T. Curtis Smith thought the essayist should have included three other conditions sometimes requiring use of forceps, viz. : Prolapse of funis, rupture of uterus, and impacted head, caused by chin parting from chest. Thought forceps were not used often as they should be; that a prejudice, profesional or otherwise, too often prevailed againt their use even in very proper cases. Was surprised at external pressure being a new thing when he saw Dr. Playfair's article ; could not remember the time when he did not use it since commencing obstetric practice; was sure he had escaped the use of forceps in some cases by using external pressure. Was aware of no authority for it, but it seemed common sense to use it, and had done so for that reason omy.

Dr. D. C. Rathburn, Sr., agreed with others that forceps were not used often enough. Thought in his earlier years he had failed to use them, sometimes where required. Approved of applying them before mother or child were endangered. Cited Seebold, of Germany, as applying them once in every —— labors; was astonished that external pressure should be considered new practice. Had used it for twenty-five years, and was instructed to do so by his lectures at college, years ago.

Dr. Barlow replied to Dr. Smith, that he believed impaction caused by chin parting from chest to be an obstetrical myth. Thought no living man had ever seen such a thing.

Dr. Train approved the paper; thought the forceps should be introduced and extracted strictly with reference to the pelvic curves, which should be the guide in their use, without regard to position of fœtal head, and that they should be resorted to only after other means of delivery had failed; thought position not always easily made out.

Dr. Hudson said position of head could nearly always be made out, even where there was thickening of scalp, etc., and attention should be paid to position of head in applying forceps as far as possible; thought the vectis too much neglected as an instrument of delivery, and to correct abnormal positions.

Dr. Smith said the position should be made out, and that some reference should be had to the head, and the forceps applied over the sides of the head; that they were more easily applied over the sides and the fœtus more readily extracted when thus applied, on account of assuming the shortest diameters, and were less apt to injure mother or child; thought the forceps a safe instrument and easily managed by skillful hands.

Dr. Reed observed that the correct position should be made out, not that we may apply the forceps more easily, but that we may the more readily imitate nature in extracting the fœtus. It is not always easy to lock the forceps. Sometimes, it will assume its place during a contraction without the effort of the operator. Contrary to usual directions, he had sometimes succeeded by using a little force of extraction or otherwise, when the forceps did not lock readily, but that they would afterward lock easily.

Dr. S. Day, of Harrisonville, being called upon, stated that in 3,000 cases of labor, he had never used the forceps; preferred to give plenty of time when necessary, and to use ergot. Thought instruments too much used; that he seldom lost mother or child without them, and preferred to give nature her strength, untrammeled by art, to the dangers of the forceps or instrumental delivery.

Dr. Hoff had seldom used the forceps, but approved of them in proper cases. Thought the mechanism of labor and forceps delivery should be well understood before attempting their use; believed they should be used to save the child, and care should be taken not to use much extractive force.

Dr. Ackley believed they were too seldom used; instead of injuring child or mother, they often protected both, where only a proper degree of force was used. Did not think great force in extraction needful, but principally the lateral force, using the forceps as a lever; to assist nature must be the object of their use.

Dr. Day, in reply to a question, said he never saw a case of exhausted uterus, and that ergot was his chief remedy in tedious labor, and where the contractions were inefficient.

Dr. Barlow could not agree with Dr. D. Thought the forceps eminently proper in some cases, and that the suffering of the mother by long waiting often laid the foundation for many serious, and sometimes incurable ills, to say nothing of the life of the child; he considered a physician in these times hardly excusable to neglect the proper means of preventing all this serious trouble; did not believe the forceps a dangerous instrument.

Dr. Smith could not understand how the gentleman had got along without instruments in some instances without losing mother and child. He had never lost a child or injured a mother by using them; thought instead of the forceps causing injury to the maternal parts or child, that more cases of sloughing were produced by long delay than by early resources to forceps; believed the forceps perfectly safe if properly used, and that they not only saved very great and prolonged suffering to the mother, but very often saved the life of the child. If we fail to do this when possible, we come short of duty.

Dr. Reed could not see how the gentleman was going to save the child without forceps when there was prolapsed funis in cases where turning or reduction could not be effected, and yet labor rather slow; believed the forceps a safe instrument and of incalculable value in the hand of a skillful accoucher. In some cases much force was actually necessary to effect delivery, but must be properly applied, and in the direction of the pelvic curves.

Dr. Hudson said cases of vesico and recto-vaginal fistula were caused by long delay ; that they were afterwards charged on the forceps.

Dr. Knight said, in 1,000 cases he had never used the forceps, but heartily approved their use in proper cases; would have used them in some cases but they were not at hand, nor could be had in time. Could not see how the forceps diminished the diameters so as to more easily effect extraction, for compression or shortening of one diameter must produce lengthening of the other ; especially would this be so if applied to the anterio-posterior diameters of the head. Thought it would be well to consider this fact before using too much compression with the forceps.

Academy adjourned to meet in Middleport, Ohio, May 25th, at 7 o'clock, P. M.—*Medical and Surgical Reporter.*

